# Determinants of the renal function after the coronary artery bypass grafting in chronic kidney disease patients: a prospective cohort study

**DOI:** 10.1186/s13019-026-04028-6

**Published:** 2026-04-15

**Authors:** Behrang Nooralishahi, Mohammad Rahmanian, Shiva Samavat, Kasra Jafari, Mohsen Nafar, Nooshin Dalili, Bahareh Haji Baratali, Reza Khademi, Mohammadsadegh Jafari, Reza Ghotbein

**Affiliations:** 1 Cardiovascular Diseases Research Institute, Tehran Heart Center, Tehran University of Medical Sciences, Tehran, Iran; 2 Student Research Committee, School of Medicine, Shahid Beheshti University of Medical Sciences, Tehran, Iran; 3 Chronic Kidney Disease Research Center, Research Institute for Urology and Nephrology, Shahid Beheshti University of Medical Sciences, Tehran, Iran; 4 Research Development Center, Arash Women’s Hospital, Tehran University of Medical Sciences, Tehran, Iran; 5 Department of Epidemiology, School of Public Health, Iran University of Medical Sciences, Tehran, Iran; 6 Department of Cardiology, Shahid Beheshti University of Medical Sciences, Tehran, Iran; 7 Student Research Committee, Faculty of Medicine, Mashhad University of Medical Sciences, Mashhad, Iran; 8 Department of Cardiovascular Diseases, Faculty of Medicine, Mashhad University of Medical Sciences, Mashhad, Iran; 9 Universal Scientific Education and Research Network (USERN), Tehran, Iran

**Keywords:** Coronary Artery Bypass, Glomerular Filtration Rate, Renal Insufficiency, Chronic, Risk Factors

## Abstract

**Background:**

This study aims to evaluate renal outcomes and identify key factors influencing estimated glomerular filtration rate (eGFR) in patients with chronic kidney disease(CKD) undergoing coronary artery bypass grafting (CABG).

**Methods:**

In a prospective cohort of 100 patients with CKD (mean age 65.5 years; 77% male; most in CKD stage 2 or 3), eGFR was measured at baseline, three months, and six months postoperatively. Generalized Estimating Equations (GEE) were applied to assess longitudinal changes in eGFR and associated predictors. Statistical analyses were performed using Stata 17.

**Results:**

Approximately 19% of patients experienced a > 30% decline in eGFR, while over 40% demonstrated improvement at six months. Advanced CKD stage, higher preoperative heart ejection fraction (EF), and longer anesthesia time were associated with greater eGFR decline. Conversely, higher preoperative hemoglobin (Hb) levels, elective surgery, and a history of cerebrovascular accident were associated with improved estimated eGFR trends.

**Conclusion:**

Several clinical and procedural factors influence postoperative renal function in CKD patients undergoing CABG. These findings support the development of targeted perioperative strategies to mitigate renal decline.

**Clinical trial registration:**

This study does not meet the criteria of a clinical trial and, therefore, was not registered in a clinical trial registry.

## Introduction

Chronic kidney disease (CKD) is a major global health issue, affecting over 800 million individuals worldwide. It is projected to become the fifth leading cause of years of life lost by 2040 [1–2]. The burden of CKD remains particularly significant in low- and middle-income countries, necessitating effective management strategies to reduce its impact [3].

Patients with CKD are at a markedly increased risk of cardiovascular diseases (CVDs), especially coronary artery disease (CAD) [4, 5]. Impaired renal function independently contributes to adverse outcomes in patients with CAD and heart failure, making CKD a critical determinant of perioperative and long-term prognosis following cardiac surgery [6–10].

Coronary artery bypass grafting (CABG) is frequently performed in patients with CKD and coexisting CAD. However, the specific trajectory of renal function following CABG in this population remains poorly characterized, with limited data on predictors of deterioration or improvement. Clarifying these effects is essential for risk stratification and guiding perioperative renal-protective strategies.

While postoperative acute kidney injury has been extensively studied, far less is known about the medium-term trajectory of renal function after CABG in patients with pre-existing CKD. Clinicians are frequently confronted with an important practical question: does kidney function recover, remain stable, or progressively decline after hospital discharge? Understanding this pattern is essential for postoperative surveillance, medication adjustment, timing of nephrology referral, and patient counseling. Therefore, evaluating longitudinal estimated glomerular filtration rate (eGFR) changes may provide clinically actionable information beyond traditional short-term acute kidney injury (AKI) definitions.

This prospective cohort study aims to evaluate longitudinal changes in renal function and to identify key predictors of eGFR trajectories in CKD patients undergoing CABG.

## Materials and methods

This prospective cohort study was conducted at Tehran Heart Center, Tehran, Iran, to evaluate renal outcomes in patients with CKD undergoing CABG. The study period extended from March 2022 to March 2023, including a six-month postoperative follow-up. Out of 146 consecutive patients screened, 46 were excluded due to end-stage renal disease or dialysis dependence before the operation (*n* = 14), previous CABG (*n* = 8), significant valvular disease (*n* = 6), hemodynamic instability (*n* = 9), or incomplete follow-up data (*n* = 9), resulting in 100 eligible participants. Inclusion criteria required patients to be at least 18 years old, diagnosed with CKD stage 1–4 according to the CKD-EPI equation, and undergoing elective or urgent isolated CABG after providing written informed consent and demonstrating willingness to complete the follow-up. Renal function was classified into stages 1 through 4 based on baseline eGFR calculated via the CKD-EPI formula.

All procedures followed a standardized institutional protocol using median sternotomy and standard cardiopulmonary bypass (CPB). Specific perioperative measures included cold blood cardioplegia, a Ringer’s-based prime solution, and a target hematocrit of 22–25% on bypass. To protect renal function, mean arterial pressure was maintained above 65 mmHg, nephrotoxic agents were avoided, and CPB time, loop diuretics, and vasopressors were minimized alongside goal-directed fluid therapy. Comprehensive data collection encompassed baseline demographics, comorbidities, medications, and intraoperative variables such as cross-clamp times and blood product transfusion. Postoperative monitoring focused on AKI per KDIGO criteria, the need for renal replacement therapy (RRT), and length of stay. Renal function was reassessed at three and six months postoperatively at a central laboratory.

Ethical approval was granted by the Research Ethics Committee of the Urology and Nephrology Research Center, Shahid Beheshti University of Medical Sciences (IR.SBMU.UNRC.REC.1400.015), and all participants provided written informed consent. Statistical analyses were conducted using Stata 17, with continuous variables reported as mean ± SD or median (IQR) and normality assessed via Shapiro-Wilk tests. The primary outcome was the longitudinal change in eGFR, analyzed using Generalized Estimating Equations (GEE) with repeated measures. Secondary outcomes included AKI incidence and eGFR decline for 30%. Covariates such as age, sex, and comorbidities were tested, with a p-value < 0.05 considered statistically significant. Analyses were performed using available-case data.

## Results

### Baseline characteristics

Of 146 screened patients, 100 met all eligibility criteria and completed follow-up. During follow-up, one patient died and one progressed to dialysis before the 3-month assessment, leaving 98 patients with available eGFR data. Between months 3 and 6, two additional deaths occurred, and one further patient was lost to laboratory follow-up. Consequently, 95 patients remained for the 6-month analysis. The mean age was 65.5 ± 8.2 years, and 77% were male. Most patients had mild-to-moderate CKD, with 46% in stage G2 and 38% in G3a or G3b. Hypertension (75%) and diabetes mellitus (43%) were the most common comorbidities. The mean preoperative eGFR was 62.5 ± 20.0 mL/min/1.73 m², and the mean ejection fraction (EF) was 44.4 ± 10.0%.

AKI developed in 50 patients (50%) during hospitalization, mostly representing stage 1 AKI. Six patients (8%) required intra-aortic balloon pump (IABP) support postoperatively. None of the patients required extracorporeal membrane oxygenation (ECMO). The median ICU stay was 5.0 days (interquartile range: 4.0–7.0 days).

Full baseline characteristics are summarized in Table 1.

**Table 1:** Clinical characteristics

Characteristic		Total valid cases (*n*)	Value
Age (years, mean ± SD)		100	65.5 ± 8.2
Sex (N, %)	male	100	77 (77%)
	female		23 (23%)
BMI (kg/m^2^, mean ± SD)		100	27.7 ± 4.9
CKD stage pre-operative (N, %)	G1	100	10 (10%)
	G2		46 (46%)
	G3a		23 (23%)
	G3b		15 (15%)
	G4		6 (6%)
Pre-operative eGFR (mL/min/1.73 m², mean ± SD)		100	62.5 ± 20.0
eGFR at three months postoperative (mL/min/1.73 m², mean ± SD)		98	58.1 ± 19.8
eGFR at six months postoperative (mL/min/1.73 m², mean ± SD)		95	58.4 ± 19.8
eGFR reduction > 30% at three months (N, %)		98	18 (≈ 18.4%)
eGFR reduction > 30% at six months (N, %)		95	18 (≈ 19%)
EF (%, mean ± SD)		98	44.4 ± 10.0
Type of surgery (N, %)	Elective	98	93(94.9%)
	Non-elective		5 (5.1%)
HTN (N, %)		100	75 (75%)
Extent of CABG (N, %)	Left main coronary artery	98	14 (14.2%)
	3 vessel		69 (70.4%)
	2 vessel		11 (11.2%)
	1 vessel		4 (4.08%)
Anemia (N, %)		94	41 (43.6%)
DM (N, %)		100	43 (43%)
CVA (N, %)		100	7 (7%)
AKI during hospitalization (N, %)		97	50 (51.5%)
RRT during hospitalization (N, %)		100	3 (3%)
Reoperation (N, %)		94	5 (5.3%)
Cardiopulmonary bypass time (minutes, mean ± SD)		95	110.9 ± 46.8
Aortic cross clamp time (minutes, mean ± SD)		95	67.4 ± 34.2
ICU stay (days, mean ± SD)		91	5.8 ± 2.9
Anesthesia time (hours, mean ± SD)		98	5.6 ± 1.3
Postoperative Intra-aortic Balloon Pump (N, %)		98	6 (≈ 6%)

Total valid case numbers vary due to death, dialysis, or missed visits

HTN: hypertension, DM: diabetes, CVA: cerebrovascular accident, AKI: acute kidney injury, RRT: renal replacement therapy, EF: ejection fraction, ICU: intensive care unit

Some percentages may not correspond exactly to whole numbers due to missing visits in specific variables. Denominator varies slightly depending on variable availability

### Risk factors for eGFR changes over time

The GEE model identified significant predictors of eGFR changes over time (Table 2).

**Fig. 1:**
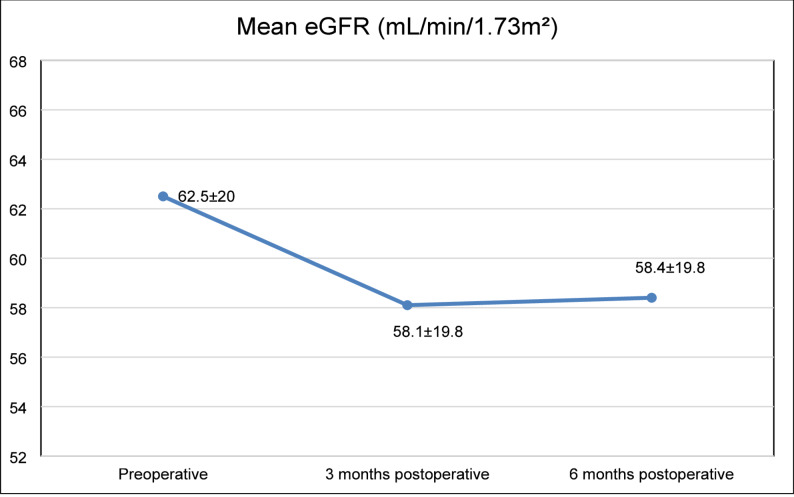
Trend of eGFR Over Time in CKD Patients Undergoing CABG. (Values are presented as mean ± standard deviation)

At three months postoperatively, 42.4% of patients experienced an improvement in eGFR, 38.4% had no significant change in eGFR, and 19.2% showed a decline in eGFR greater than 30%. At six months postoperatively, 40.8% of patients demonstrated improved eGFR, 39.8% remained stable, and 19.4% experienced a decline in eGFR of 30% or more. eGFR declined modestly in the first 3 months postoperatively and remained relatively stable thereafter (Fig. 1).

As expected in a longitudinal model, eGFR values at 3 and 6 months were lower than baseline. On average, eGFR decreased by 7.011 mL/min/1.73 m² at three months and 7.628 mL/min/1.73 m² at six months after surgery compared to eGFR before surgery (*p* < 0.001 for both).

Preoperative hemoglobin level was identified as a protective factor. Higher preoperative hemoglobin (Hb) levels were associated with higher eGFR measurements during follow-up (β = +1.85 per 1 g/dL), indicating a more favorable renal trajectory. A history of cerebrovascular accident (CVA) was unexpectedly associated with a favorable eGFR trajectory (coefficient: +10.25; *p* = 0.035). Elective surgery was associated with better renal outcomes than urgent surgery (coefficient: +10.93; *p* = 0.036).

Conversely, more advanced CKD stages (G3a, G3b, and G4) were significantly associated with greater postoperative eGFR decline compared to stage G1, with coefficients of -15.509, -27.646, and − 33.946, respectively (*p* < 0.001 for all). Additionally, longer anesthesia time was associated with worse renal outcomes (coefficient: -2.573 per hour; *p* = 0.040).

A higher EF was paradoxically associated with greater decline in eGFR (coefficient: -0.24 per 1% increase in EF; *p* = 0.017). This unexpected relationship warrants further exploration to elucidate the underlying mechanisms.

Other variables, including age, AKI, RRT, diabetes, and hypertension, were not significantly associated with long-term eGFR change. Full details are provided in Table 2.

**Table 2:** Results of GEE analysis on predictors of GFR trend

Predictor Variables		β Coefficient	SE	*p*-value	95% CI	
					Lower	Upper
Time (Pre-operative GFR as reference)	3 months post-operative	-7.011	1.720	0.001	-10.383	-3.639
	6 months post-operative	-7.628	1.738	0.001	-11.035	-4.221
Sex (male as reference)		-1.774	3.257	0.586	-8.157	4.609
HTN (no as reference)		-0.078	2.372	0.974	-4.728	4.571
DM (no as reference)		4.408	2.467	0.074	-0.426	9.242
CVA (no as reference)		10.250	4.854	0.035	0.737	19.763
Surgery Type (non-elective as reference)		10.934	5.212	0.036	0.719	21.149
Smoking (no as reference)		-0.404	4.194	0.923	-8.625	7.816
AKI During Admission (no as reference)		3.715	2.651	0.161	-1.481	8.912
RRT During Admission (no as reference)		-4.455	5.753	0.439	-15.731	6.822
Anemia (no as reference)		0.006	2.963	0.998	-5.802	5.813
Reoperation (no as reference)		1.903	5.100	0.709	-8.092	11.898
Stage of CKD (G1 as reference)	G2	-0.277	3.760	0.941	-7.647	7.092
	G3a	-15.509	4.027	0.001	-23.402	-7.616
	G3b	-27.646	4.958	0.001	-37.364	-17.929
	G4	-33.946	5.408	0.001	-44.545	-23.348
Age		-0.219	0.132	0.097	-0.477	0.039
BSA		4.234	6.435	0.511	-8.378	16.847
EF		-0.238	0.100	0.017	-0.434	-0.042
Preoperative Hb		1.852	0.674	0.006	0.532	3.172
Aortic Cross Clamp Time (minutes)		0.130	0.086	0.132	-0.039	0.299
Cardiopulmonary Bypass (minutes)		-0.052	0.066	0.430	-0.180	0.077
Anesthesia Time (hours)		-2.573	1.253	0.040	-5.029	-0.117
ICU Stay		0.112	0.597	0.851	-1.058	1.283

HTN: hypertension, DM: diabetes, CVA: cerebrovascular accident, AKI: acute kidney injury, RRT: renal replacement therapy, op: operation, BSA: body surface area, EF: ejection fraction, Hb: hemoglobin, SE: standard error, CKD: chronic kidney disease

## Discussion

This study assessed renal function trajectories in patients with CKD undergoing CABG and identified key perioperative predictors of eGFR changes over a six-month period. Our findings confirm that the severity of baseline CKD is a major determinant of postoperative renal decline, and also reveal several unexpected associations that merit further investigation.

### Main findings

An advanced preoperative CKD stage was strongly associated with a stepwise and statistically significant decline in eGFR following CABG. Patients in stages G3a, G3b, and G4 experienced the greatest reductions in renal function over time, underscoring the need for tailored risk stratification and intensified perioperative management in this subgroup.

Although over 50% of patients developed AKI postoperatively—predominantly stage 1 AKI defined by minimal serum creatinine increases—this was not significantly associated with long-term changes in eGFR. This may reflect the transient and reversible nature of mild AKI episodes, or that perioperative interventions effectively mitigated their long-term impact. In contrast to studies linking AKI to increased cardiovascular risk and mortality [11] our findings suggest that early-stage AKI in the context of closely monitored care may not always predict chronic renal decline. However, a longer follow-up is needed to fully evaluate this relationship.

Unexpectedly, higher baseline EF was associated with greater eGFR decline, contrary to prior reports that link reduced EF with postoperative renal impairment [12, 13, 14]. One hypothesis is that patients with lower EF were more aggressively managed perioperatively with closer hemodynamic monitoring and stricter fluid control, possibly buffering their renal function. An additional hypothesis may relate to differences in chronic hemodynamic adaptation. Patients with long-standing reduced EF may tolerate relative reductions in perfusion pressure differently than those with preserved EF. In such circumstances, standardized intraoperative Mean arterial pressure (MAP) targets may not account for individual patient variations in baseline perfusion pressure. Alternatively, this unexpected association should be interpreted cautiously. Given the relatively small sample size, the finding may reflect residual confounding, selection mechanisms, or random variation rather than a true causal physiological relationship. Therefore, EF should not yet be considered a reliable predictor of long-term renal outcomes without confirmation in larger cohorts. Future studies incorporating preoperative blood pressure values, time-weighted MAP, and perfusion variability may help clarify this mechanism.

Patients undergoing non-elective CABG exhibited a higher risk of postoperative renal complications, consistent with previous research associating urgent CABG with greater renal, cardiac, and respiratory complications, prolonged hospital stays, and increased mortality (15). The increased risk may be attributed to hemodynamic instability, greater preoperative renal dysfunction, and the use of vasopressors and nephrotoxic agents in emergency settings.

Higher preoperative Hb levels were significantly associated with more favorable renal trajectories. This aligns with prior studies showing that preoperative anemia increases the risk of AKI and other adverse outcomes after cardiac surgery [16]. Improved oxygen delivery and reduced ischemic burden may underlie this protective effect.

Another novel finding was the association between a history of cerebrovascular accident (CVA) and improved postoperative renal function. This may reflect selection or care bias: patients with CVA history may have received more intensive intraoperative and postoperative monitoring due to perceived higher risk. However, given the lack of direct physiological explanation and the small number of patients with CVA (*n* = 7), this association should be interpreted cautiously and warrants confirmation in larger studies.

Longer anesthesia duration was also associated with worse renal outcomes, consistent with existing literature linking prolonged operative time and cardiopulmonary bypass to renal injury [17]. Minimizing anesthesia and bypass duration may therefore be a viable target for renal-protective strategies.

### Strength and limitations

The strengths of our study include a prospective design, structured follow-up, and use of GEE to model renal trends over time. Our dataset allowed for adjustment of multiple confounders and included a wide range of pre-, intra-, and postoperative variables.

However, the study has limitations. First, the single-center design and modest sample size limit generalizability. Second, although we adjusted for known confounders, unmeasured factors such as medication changes or fluid shifts may have influenced renal outcomes. Third, the six-month follow-up period may be insufficient to capture delayed eGFR deterioration. Finally, while we documented AKI, we did not classify severity beyond stage 1 or evaluate biomarkers of kidney injury.

### Implications for future research

Future studies should:

- Enroll larger, multicenter cohorts for external validation.
- Extend follow-up duration to assess long-term renal and cardiovascular outcomes.
- Incorporate cardiac function measures, imaging, and cardiac enzyme levels (including troponin and NT-proBNP) in follow-up to assess cardiorenal interactions.
- Explore perioperative interventions—such as hemoglobin optimization or anesthesia time reduction—as potential targets to preserve renal function.

## Conclusion

This prospective study highlights the significant role of preoperative CKD stage in determining renal outcomes following CABG. Patients with more advanced CKD (stages G3a, G3b, and G4) were significantly more likely to experience postoperative eGFR decline over six months.

Notably, higher preoperative hemoglobin levels, elective surgery, and a history of CVA were associated with more favorable renal trajectories, while higher EF and longer anesthesia time predicted greater renal decline. These findings suggest that both baseline characteristics and modifiable perioperative factors contribute to long-term renal outcomes in this population.

Although postoperative AKI was common, it was not independently associated with long-term eGFR decline, particularly when limited to stage 1 AKI. This underscores the need to interpret mild AKI episodes with caution and consider their clinical context.

Targeted preoperative optimization—especially hemoglobin correction, surgical planning, and careful intraoperative management—may improve renal outcomes in patients with CKD undergoing CABG. Further multicenter studies with longer follow-up are warranted to validate these findings and inform risk-reduction strategies.

## Data Availability

The datasets used and/or analyzed during the current study are available from the corresponding author on reasonable request.

## Abbreviations

AKI: Acute kidney injury
BMI: Body mass index
CABG: Coronary artery bypass Grafting
CAD: Coronary artery disease
CKD: Chronic kidney disease
CKD-EPI: Chronic kidney disease epidemiology collaboration
CPB: Cardiopulmonary bypass
CRF: Case report form
CVA: Cerebrovascular accident
CVD: Cardiovascular disease
EF: Ejection fraction
eGFR: Estimated glomerular filtration rate
GEE: Generalized estimating equations
HTN: Hypertension
Hb: Hemoglobin
ICU: Intensive care unit
LVEF: Left ventricular ejection fraction
MAP: Mean arterial pressure
RRT: Renal replacement therapy
SBMU: Shahid Beheshti medical university
THC: Tehran heart center
